# The smoking-dyslipidaemia dyad: A potent synergistic risk for atherosclerotic coronary artery disease

**DOI:** 10.1177/2048004020980945

**Published:** 2021-03-15

**Authors:** Vineet Prakash, Sams Jaker, Amjad Burgan, Adam Jacques, David Fluck, Pankaj Sharma, Christopher H Fry, Thang S Han

**Affiliations:** 1 Department of Radiology, Ashford & St Peter’s Foundation Trust, Chertsey, UK; 2 Diagnostic Imaging, Royal Surrey County Hospital, Guildford, UK; 3 Department of Cardiology, Ashford & St Peter’s Foundation Trust, Chertsey, UK; 4 Institute of Cardiovascular Research, Royal Holloway University of London, Egham, UK; 5 School of Physiology, Pharmacology and Neuroscience, University of Bristol, Bristol, UK

**Keywords:** Atherosclerosis, coronary artery calcium score, myocardial infarct, ischaemic heart disease

## Abstract

**Background:**

Smoking and dyslipidaemia are known individual risk factors of coronary artery disease (CAD). The present study examined the combined risk of smoking and dyslipidaemia on coronary atherosclerosis.

**Methods:**

Coronary artery calcium (CAC), measured by cardiac CT, was used to assess the extent of CAD, which was related to smoking and dyslipidaemia using logistic regression, adjusted for age, sex, hypertension, BMI and family history of ischaemic heart disease.

**Results:**

Seventy-one patients (46 men, 25 women: median age of 53.7yrs; IQR = 47.0–59.5) were recruited. The mean log_10_ CAC score in never-smokers without dyslipidaemia (reference group) was 0.37 (SD = 0.73), while the value in those with a history of smoking was 0.44 ± 0.48 (mean difference: 0.07, 95%CI:–0.67 to 0.81, *p* = 0.844), dyslipidaemia was 1.07 ± 1.08 (mean difference: 0.71, 95%CI: 0.24 to 1.17, *p* = 0.003), and both risk factors was 1.82 ± 0.64 (mean difference: 1.45, 95%CI:0.88 to 2.02, *p* < 0.001). For individuals in the reference group, the proportions with none, one and multiple vessel disease were 80.6%, 16.1% and 3.2%; for those with a history of smoking or with dyslipidaemia were 50.0%, 25.0% and 25.0%; and for those with both risk factors were 8.3%, 25.0% and 66.7%. Patients with a history of both risk factors had greater adjusted risks of having one- vessel disease - OR = 14.3 (95%CI = 2.1–98.2) or multiple vessel disease: OR = 51.8 (95%CI = 4.2–609.6).

**Conclusions:**

Smoking and dyslipidaemia together are associated with high coronary artery calcification and CAD, independent of other major risk factors.

## Introduction

Acute myocardial infarction (AMI) and sudden cardiac death, as a consequence of coronary artery disease (CAD), are among the leading causes of mortality in the UK^1^ and US.^2^ The underlying reason for these conditions mostly arises from acute intraluminal coronary thrombus formation that completely or almost completely occludes an epicardial coronary artery.^3^ The pathogenesis of AMI begins with the progressive development of atherosclerosis which gradually thickens the inner layer of the coronary arteries leading to narrowing of the arterial lumen.^4,5^ Atherosclerosis, considered to be a low-grade inflammatory state, is accelerated by risk factors such as chronic cigarette smoking,^6,7^ dyslipidaemia,^8,9^ hypertension,^10^ diabetes^9,11^ and genetic predisposition.^12^

 Normally, CAD is assessed by percutaneous coronary angiography, which poses a number of hazards to patients such as contrast medium-induced acute kidney injury,^13^ thyroid dysfunction^14^ and pseudoaneurysms at the puncture site.^15^ Increasingly, less-invasive cardiac computerised tomography (CT) used to measure coronary artery calcium (CAC) has been used to detect vessel blockage by plaque accumulation. Since coronary atherosclerosis is the only condition associated with calcium deposition in the coronary arteries,^16^ CAC is a reliable indicator of atherosclerosis, *i.e.* CAD,^17^ itself highly predictive of future cardiovascular events.^18,19^ Conversely, the absence of CAC is highly specific in excluding obstructive coronary artery stenosis.^16^ Cardiac CT has been recommend by the National Institute for Health and Care Excellence (NICE) as the first-line investigation in patients presenting with stable chest pain with low-to-intermediate predicted risk of CAD.^20^

Although smoking and dyslipidaemia are known individual risk factors of CAD, there is a lack of information on their combined effects on CAC deposition. This study examined the extent of atherosclerosis, indicated by CAC measurement, in patients with a history of cigarette smoking and dyslipidaemia compared with subjects free of these risk factors.

## Methods

We retrospectively collected baseline information on 71 patients, who met the recommended NICE criteria for cardiac CT for investigations of stable chest pain with low-to-intermediate predicted risk of CAD,^20^ between November 2015 and July 2016 in a single National Health Service hospital.

### Data collection

Data collected included demographic factors (age and sex), history of smoking, pre-existing co-morbidities including dyslipidaemia, hypertension, diabetes, family history of ischaemic heart disease (IHD) and medications (antiplatelets, HMG-CoA reductase inhibitors, anti-hypertensive and antianginal drugs). Lipid profile, blood pressure, weight and height and BMI (kg/m^2^), measured at the time of investigations were recorded.

### Cardiac CT and quantification of CAC and vessel disease

Dedicated cardiac CT (Aquilion PRIME, Japan) using a single-source helical CT scanner with 80 detectors was used to detect the presence, location and extent of calcified plaque in the coronary arteries. A CAC score was calculated using a post-processing workstation (Vitrea FX, version 1.0, Vital Images, Minnetonka, USA), with calcium score analysis software (VScore, Vital Images). Coronary calcium was defined as an area of at least three ‘face-connected’ voxels in the axial plane in the course of a coronary artery, with an attenuation threshold-value of ≥130 Hounsfield units (HU).^21^ Three in-axial plane face-connected voxels correspond to a minimum lesion area >1 mm^2^ that is used as reference value in calcium scores.

The presence and quantity of CAC was evaluated based on the Agatston scoring method.^22^ The CAC score was calculated as the product of the area of calcification by an attenuation factor based on peak plaque density. Coronary artery-specific scores were obtained for the three major coronary arteries (left anterior descending, left circumflex, and right coronary artery) and the total calcium score from these three arteries were summated for the analysis. The CAC score was used to represent identifiable plaque burden.^18^ CAC scores were not be normally distributed and showed a wide range of values. Thus, data were transformed to log_10_ values (log_10_ CAC) and these are used as values for analysis.

### Categorisation of main outcome variables

Smoking status was grouped into never-smokers and current/former smokers. Dyslipidaemia was defined as those known to have a diagnosis of dyslipidaemia or treated with an antihyperlipid agent or with total cholesterol >5.18 mmol/l (200 mg/dl) or fasting triglycerides ≥1.7 mmol/l (150 mg/dl). BMI was categorized into two groups at a cut-off of 25 kg/m^2^. Data for underlying co-morbidities were dichotomised according to the absence or presence of the condition. The extent of CAD was first assessed by individual vessel disease (0, 1, 2, and 3) followed by categorisation into three groups: 1) no vessel disease, 2) single vessel disease, and 3) multiple vessel disease (2 or 3). Smoking status and dyslipidaemia were categorised into three groups: 1) no history of smoking or dyslipidaemia, 2) either history of smoking or dyslipidaemia, and 3) history of smoking and dyslipidaemia. Hypertension status was defined as normotensive (no history of hypertension), controlled hypertension (hypertension history with systolic blood pressure <140 and diastolic blood pressure <90 mmHg), and uncontrolled hypertension (hypertension history with raised systolic blood pressure ≥140 or diastolic blood pressure ≥90 mmHg).^23,24^

### Statistical analysis

CAC values are quoted as medians with interquartiles (IQR) as the data sets were positively skewed. These values were also logarithmically transformed (log_10_ CAC) for multivariate analysis: both are quoted in the text for convenience. Differences in CAC values between two groups of risk factors were assessed by non-parametric (Mann-Whitney) independent *t*-tests, and group differences were examined by Kuskal-Wallis H tests. For log_10_ CAC data equivalent comparisons used Student t-tests and analysis of variance (ANOVA) with *post hoc* LSD tests to determine significant differences. The prevalence of patients with risk factors (history of smoking and dyslipidaemia) within each category of CAD was assessed by chi-squared tests. Multivariable regression was conducted to assess the association of the history of smoking and dyslipidaemia with the extent of CAD (1 or ≥2 coronary vessel disease), adjusted for age, sex, hypertension and family history of IHD. The present study had only two patients with diabetes therefore was not used as confounding factor. Data were analysed using BM SPSS Statistics, v25.0 (IBM Corp., Armonk, NY). The null hypothesis was rejected when *p* < 0.05.

## Results

Seventy-one patients were recruited with median age (IQR) 53.7 yr (47.0–59.5): 31 patients (43.7%) had no history of smoking or dyslipidaemia; 28 (39.4%) with either history of smoking or dyslipidaemia; and 12 (16.9%) a history of smoking and dyslipidaemia. There were 44 patients (62%) with normotension, 12 (16.9%) with controlled hypertension and 15 (21.1%) with uncontrolled hypertension. The mean (±SD) log_10_ CAC score was 0.84 ± 0.98 (range 0–3.07). After cardiac CT, 71.8% of patients were discharged and 28.2% were followed up for further evaluation. There were 22.5% of patients taking an antiplatelet or statin and 33.8% on cardiac medication (Table 1). Compared with patients who were followed up for further evaluation, there were fewer patients who were discharged who were being treated with an antiplatelet agent (55.0% versus 9.8%, χ^2^ =16.8, *p* < 0.001), statin (45.0% versus 13.7%, χ^2^ = 8.0, *p* = 0.007), cardiac medications (50.0% versus 27%, χ^2^ = 3.3, *p* = 0.065), and any of these medications (33.3% versus 65.0%, χ^2^ =5.9, *p* = 0.015).

**Table 1.** Subject characteristics.

	Median	(IQR)
Age (years)	53.7	47.0–59.5
BMI (kg/m^2^)	26.5	23.9–29.9
Total cholesterol (mmol/l)	5.35	4.23–6.08
Triglycerides (mmol/l)	1.1	1.0–1.9
Systolic blood pressure (mmHg)	135	118–148
Diastolic blood pressure (mmHg)	82	71–90
HbA_1C_ (mmol/mol)	37	34–40
	n	%
Men: women	46: 25	64.8: 35.2
BMI ≥25	47	66.2
History of smoking (current /former smokers)	18	25.4
History of dyslipidaemia	34	47.9
No history of smoking or dyslipidaemia	31	43.7
History of smoking	6	8.5
History of dyslipidaemia	22	31.0
History of smoking and dyslipidaemia	12	16.9
History of diabetes	2	2.8
No history of hypertension	44	62.0
History of hypertension with controlled BP (SBP <140 mmHg and DBP <90 mmHg)	12	16.9
History of hypertension with uncontrolled BP (SBP ≥140 mmHg or DBP ≥90 mmHg)	15	21.1
Family history of IHD	34	47.9
Number of vessel disease		
0: 1: 2: 3	41: 15: 7: 8	57.7: 21.1: 9.9: 11.3
≥1	30	42.3
≥2	16	22.5
Follow up: discharge post-cardiac CT investigation	20: 51	28.7: 71.8
Treatment post-cardiac CT investigation		
Antiplatelets	16	22.5
HMG-CoA reductase inhibitors	16	22.5
Antihypertensive/cardiac medications^†^	24	33.8
Any of the three drugs above	30	42.3

^†^Angiotensin-converting enzyme inhibitors, angiotensin receptor blockers, beta-blockers, calcium channel blockers or diuretics; BP, blood pressure; SBP, systolic blood pressure; DBP, diastolic blood pressure.

**Figure 1.**
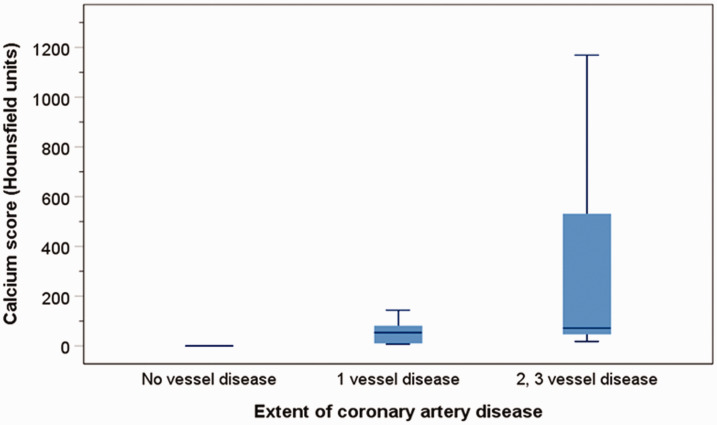
Boxplots of the median and interquartile ranges and whiskers represent the 5th and 95th percentiles of CAC scores in relation to the extent of coronary artery disease.

The CAC score progressively and significantly increased with the extent of CAD (Kuskal-Wallis H test: *p* = 0.034), with a median score of zero for no vessel disease, rising to a median CAC score of 54 HU (IQR = 12–84) for 1 vessel disease, and 71.5 HU (IQR = 47.3–529) for two or three vessel disease (Figure 1). Compared to those without coronary vessel disease, the log_10_ CAC score was higher in patients with one vessel disease by 1.51– (95% CI = 1.29 to 1.73, *p* < 0.001), and in those with two or three vessel disease by 2.03 (1.82 to 2.25, *p* < 0.001). In addition, the log_10_ CAC score in those with 2 or 3 vessel disease was significantly greater than those with one vessel disease (*p* < 0.001).

Furthermore, log_10_ CAC score was significantly higher in those with a history of dyslipidaemia than those without: mean difference = 0.96 (0.55 to 1.36, *p* < 0.001) and in those with a history of smoking than those who never smoked: mean difference = 0.70 (0.19 to 1.21, *p* = 0.008). There were no significant differences in log_10_ CAC score between those with a history of hypertension or normotension, BMI below or above 25 kg/m^2^, or with or without family history of IHD (Table 2).

**Table 2.** Coronary artery calcium score in patients with different risk factors.

	Differences in log_10_ CAC score		
	Mean difference	95% CI	p
Smoking *vs* never-smokers	0.70	0.19 to 1.21	0.008
Dyslipidaemia *vs* no dyslipidaemia	0.98	0.55 to 1.36	<0.001
Hypertension *vs* normotension	0.44	–0.03 to 0.90	0.067
Family history of IHD *vs* no family history of IHD	–0.42	–0.87 to 0.04	0.071
BMI <25 *vs* BMI ≥25	0.36	–0.12 to 0.84	0.143
0 *vs* ≥1 vessel disease	1.73	1.51 to 1.95	<0.001
0 *vs* ≥2 vessel disease	1.62	1.23 to 2.02	<0.001

IHD, ischaemic heart disease; BMI, body mass index.

In patients who had: no history of hypertension; controlled hypertension; and uncontrolled hypertension, the respective median (IQR) CAC scores were 0 (0–24.8), 0 (0–14.8), and 54 (26–84) HU respectively. The corresponding mean log_10_ CAC values were 0.67, 0.54 and 1.56 (ANOVA for group differences: *F* = 5.1, *p* = 0.004). *Post hoc* tests, conducted using the least significant differences (LSD) method showed log_10_ CAC scores of the uncontrolled hypertensive group were higher than that of controlled hypertensive by 1.02 (95%CI = 0.32–1.73, *p* = 0.005) and that of normotensive group by 0.89 (0.35–1.44, *p* = 0.002). There were no significant differences in log_10_ CAC between normotensive and controlled hypertensive groups (*p* = 0.656).

Among the 34 patients with a history of dyslipidaemia, there were 16 (47.1%) patients taking a statin. The median CAC score was 36 HU (IQR = 3–529) compared with the CAC score of 48.5 (0–71.5) for those who were not taking a statin and were not significantly different, *p* = 0.366.

**Figure 2.**
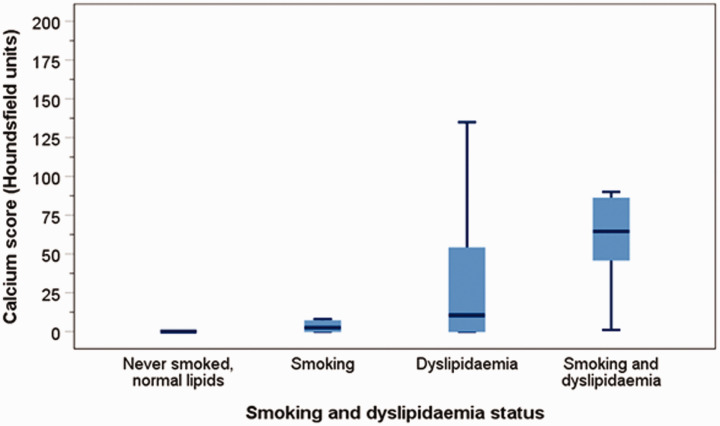
Boxplots of the median and interquartile ranges and whiskers represent the 5th and 95th percentiles of CAC scores in different status of smoking and dyslipidaemia.

The CAC score also progressively and significantly increased with smoking and dyslipidaemia (Kuskal-Wallis H test: *p* < 0.001). Among those without a history of smoking or dyslipidaemia, the CAC score was zero, rising to a median of 2.5 (IQR = 0–7.3) for those with a history of smoking, 10.5 (0–61.5) for dyslipidaemia, and to 64.5 (46–88) for those with a history of both smoking and dyslipidaemia (Figure 2). ANOVA showed an increase of mean log_10_ CAC scores from the reference group to patients with one risk factor (smoking or dyslipidaemia) and then to those with both risk factors (F = 10, p < 0.001). However, *post hoc* LDS tests showed that the increase of mean log_10_ CAC scores just failed to reach statistical significance with one risk factor, whereas significance was achieved with both risk factors. Log_10_ CAC scores rose progressively from the lowest value among those without a history of smoking or dyslipidaemia (reference): 0.37 ± 0.73 to those with a history of smoking: 0.44 ± 0.48 (mean difference: 0.07, 95%CI: -0.67 to 0.81, *p* = 0.844), dyslipidaemia: 1.07 ± 1.08 (mean difference: 0.71, 95%CI: 0.24 to 1.17, *p* = 0.003), and to those with a history of both smoking and dyslipidaemia: 1.82 ± 0.64 (mean difference: 1.45, 95%CI: 0.88 to 2.02, p < 0.001). The CAC score was significantly higher in those with a history of both smoking and dyslipidaemia than in those with a history of either smoking or dyslipidaemia (mean difference: 0.88, 95%CI: 0.30 to 1.46, *p* = 0.003).

**Figure 3.**
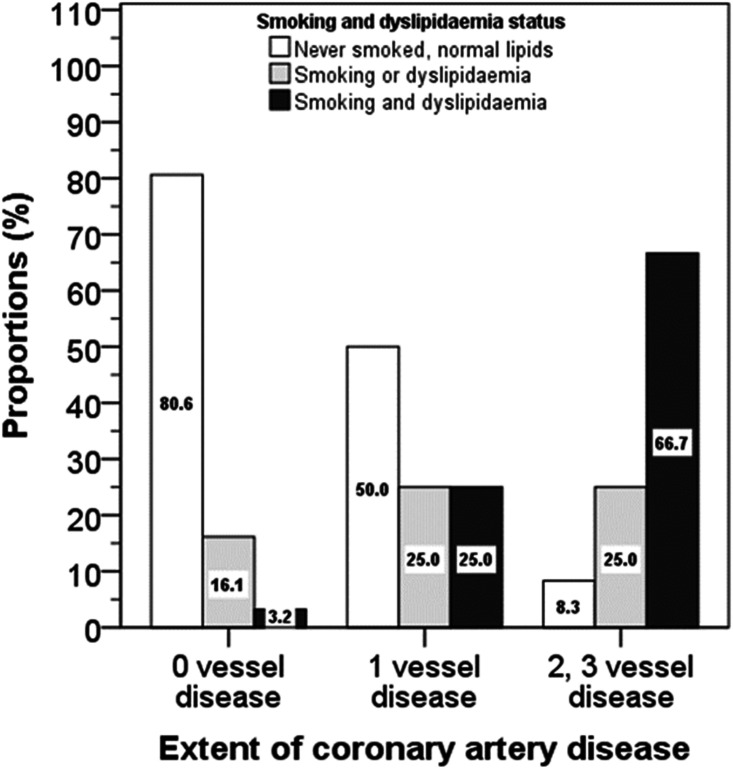
Proportions of patients with CAD in relation to smoking and dyslipidaemia status. White bars = 0 vessel disease, grey bars = 1 vessel disease and black bars = 2 or 3 vessel disease (χ^2^ = 24.6, p < 0.001).

For patients with no vessel disease 80.6% had no history of smoking or dyslipidaemia, whilst 16.1% either smoked or had dyslipidaemia, whilst 3.2% had both risk factors. For those with 1-vessel disease corresponding percentages were 50.0, 25.0 and 25.0% and for patients with ≥2-vesel disease these were 8.3, 25.0 and 66.7% (χ^2^ = 24.6, *p* < 0.001) (Figure 3).

Compared with patients without a history of smoking or dyslipidaemia, those with a history of either smoking or dyslipidaemia had increased risk of having at least 1-vessel disease: unadjusted OR (95% CI) = 4.2 (1.3–13.3, *p* = 0.016), and age, sex, hypertension status and family history of IHD adjusted OR = 4.8 (1.3–18.7, *p* = 0.020). For those with ≥2-vessel disease: unadjusted OR = 10.0 (1.1–87.4, *p* = 0.037), and adjusted OR = 9.8 (1.1–89.7, *p* = 0.043). Among those who both smoked and had dyslipidaemia, the risk was further increased for having at least one coronary vessel disease: unadjusted OR = 20.8 (3.6–121.1, *p* = 0.001), adjusted OR = 14.3 (2.1–98.2, *p* = 0.001), and for 2 or 3 vessel disease: unadjusted OR = 60.0 (5.9–614.2, *p* = 0.001), adjusted OR = 51.8 (4.4–609.6, *p* = 0.002) (Table 3).

**Table 3.** Logistic regression examining the association of smoking and dyslipidaemia with the extent of coronary artery disease.

	Risk of coronary artery disease					
	Unadjusted			Adjusted for age, sex, family history of IHD and hypertension status^†^		
	OR	95% CI	*p*	OR	95% CI	*p*
At least 1 vessel disease						
Never smoked and normal lipids (reference)	1	–	–	1	–	–
Smoking or dyslipidaemia	4.2	1.3–13.1	0.016	4.8	1.3–17.7	0.020
Smoking and dyslipidaemia	20.8	3.6–121.1	0.001	14.3	2.1-98.2	0.007
≥2 vessel disease						
Never smoked and normal lipids (reference)	1	–	–	1	–	–
Smoking or dyslipidaemia	10.0	1.1–87.4	0.037	9.8	1.1–89.7	0.043
Smoking and dyslipidaemia	60.0	5.9–614.2	0.001	51.8	4.4–609.6	0.002

IHD, ischaemic heart disease.

^†^Hypertension status: no history of hypertension, controlled hypertension and uncontrolled hypertension.

## Discussion

This study of individuals undergoing investigations for stable chest pain showed that about 80% of those without a history of smoking or dyslipidaemia had no evidence of atherosclerotic CAD based on CAC measured by CT. Among those with a history of smoking or dyslipidaemia, more than half had CAD, whilst two-thirds of all patients with both risk factors had CAD. These patients had an eight-fold increase in having at least one coronary vessel disease compared with those without a history of smoking and dyslipidaemia, independent of age, sex and major cardiometabolic risk factors. The synergistic effects of cigarette smoking and dyslipidaemia shown here emphasise the interaction between these two major risk factors in the pathogenesis of coronary atherosclerosis. Prevention of these two risk factors is essential to reduce the development of CAD and its adverse health consequences.

Our study showed smoking alone does not increase CAC scores more than never smokers with normal lipids, but there were only six patients (8.5%) in the sample. The association between smoking and CAC scores have been reported in previous studies,^25^ but there remains uncertainty of the underlying pathophysiological mechanisms of atherosclerosis but it is possible that atherosclerosis is promoted by toxic products in tobacco.^12^ Different compounds in cigarette smoke have the potential to activate endothelial NADPH oxidase^26^ and cause mitochondrial oxidative stress,^27^ while increased production of superoxide and peroxynitrite leads to vascular inflammation and irreversible DNA damage.^28,29^

Other known risk factors of atherosclerosis formation have been established including dyslipidaemia, hypertension and diabetes.^30^ Similar to the adverse effects of smoking, dyslipidaemia was also associated with raised CAC scores and increased risk of CAD in the present study, also consistent with previous studies.^31^ Hypercholesterolaemia and the development of atherosclerosis is well established and has been the subject of intense research.^32^ Whilst the majority of published studies have focused on individual risk factors of CAD,^33^ far fewer studies have been conducted to examine any combined effects of smoking and dyslipidaemia on the development of CAD. This is surprising given that smoking and dyslipidemia are two different cardiovascular risk factors that can have individual impacts on CAC scores. A study of patients with type-2 diabetes found that smokers had increased levels of serum TC and triglycerides, lower HDL-C and HDL2-C, and increased hepatic lipase activity – an early sign of atherosclerosis – and it was suggested that accentuation of dyslipidaemia by smoking may be driven by increased hepatic lipase activity.^34^ It is possible that inflammation of the coronary vessel caused by one of these risk factors exposes the weakened vessel to other risk factor to cause further damage. The severity of abdominal aortic aneurysm was accelerated by cigarette smoke in hypertensive mice,^35^ although we showed here that hypertension did not increase the risk of CAD in those with history of dyslipidaemia or smoking.

We found no associations of hypertension, family history of IHD or BMI with CAC or with the extent of CAD. It is possible that hypertension and those with family history of IHD may have been appropriately treated to prevent the development of atherosclerosis while BMI was relatively low overall. Previous studies have shown that a high CAC score in patients with diabetes increased the risk of CVD-dependent mortality.^36^ Therefore it would be of interest to analyse the contribution of altered glucose metabolism to CAC but our data included only two cases with diabetes.

New guidelines have been published recently by The Task Force for the management of dyslipidaemias, under the auspices of the European Society of Cardiology and European Atherosclerosis Society.^37^ The initial approach was to assess the 10-year risk of fatal CVD for European populations at low CVD risk using the Systematic Coronary Risk Estimation chart. The risk factors are based on age, gender, smoking, systolic blood pressure, and total cholesterol. Primary prevention includes promotion of health lifestyle including: a healthy diet low in saturated fat with predominant proportions of wholegrain products, vegetables, fruit, and fish; regular exercise of 3.5–7 hours of moderately vigorous physical activity a week, or 30–60 min each day. These should aim to keep the body mass index in the range of 20–25 kg/m^2^, a waist circumference <94 cm in men and <80 cm in women, as well as blood pressure <140/90 mmHg, while exposure to any form of tobacco should be avoided. Secondary prevention with a drug in conjunction with lifestyle modification should be considered if the level of risk is high. There are different approaches to smoking cessation for patients with CVD, including a number of pharmacological agents such as varenicline, nicotine replacement therapy, bupropion SR which may be used as single or dual therapy.^38^

### Strengths and limitations

The present study contained a cohort of patients undergoing investigations for chest pain using a non-invasive technique, which has only been introduced into clinical practice quite recently. The data showed reliable results in the correlations of CAC obtained by CT with risk factors and with the extent of CAD. The present study combined current smokers with former smokers based on evidence that both groups are at increased risk of coronary atherosclerosis.^39^ However, it is limited by the retrospective cross-sectional design which does not allow inferences on causality of risk factors and CAD. Because of the small sample size, the relatively large odds ratio values and wide confidence intervals means they should be interpreted with caution. It would be expected these values should diminish with larger sample numbers. We found that among patients with a history of dyslipidaemia, the CAC score in those who were treated with a statin did not differ from that in untreated individuals. However, it should be recognised that an overestimation of CAC score in this group may occur because statins are known to promote calcification of the atherosclerotic plaque.^40^

In conclusion, smoking and dyslipidaemia are both associated with high coronary artery calcification and CAD, independent of other major risk factors. Prevention of smoking and tighter control of lipid levels are important to minimise future symptoms of CAD.

## Provenance

Invited contribution.
